# Supplementary material for: Combination of Resminostat with Ruxolitinib Exerts Antitumor Effects in the Chick Embryo Chorioallantoic Membrane Model for Cutaneous T Cell Lymphoma
Source: Cancers (Basel). 2022 Feb 20;14(4):1070. doi: 10.3390/cancers14041070 (PMC8870185; doi:10.3390/cancers14041070)
Supplement: Supplementary file 1 [file cancers-14-01070-s001.zip › cancers-1591577-supplementary.pdf]

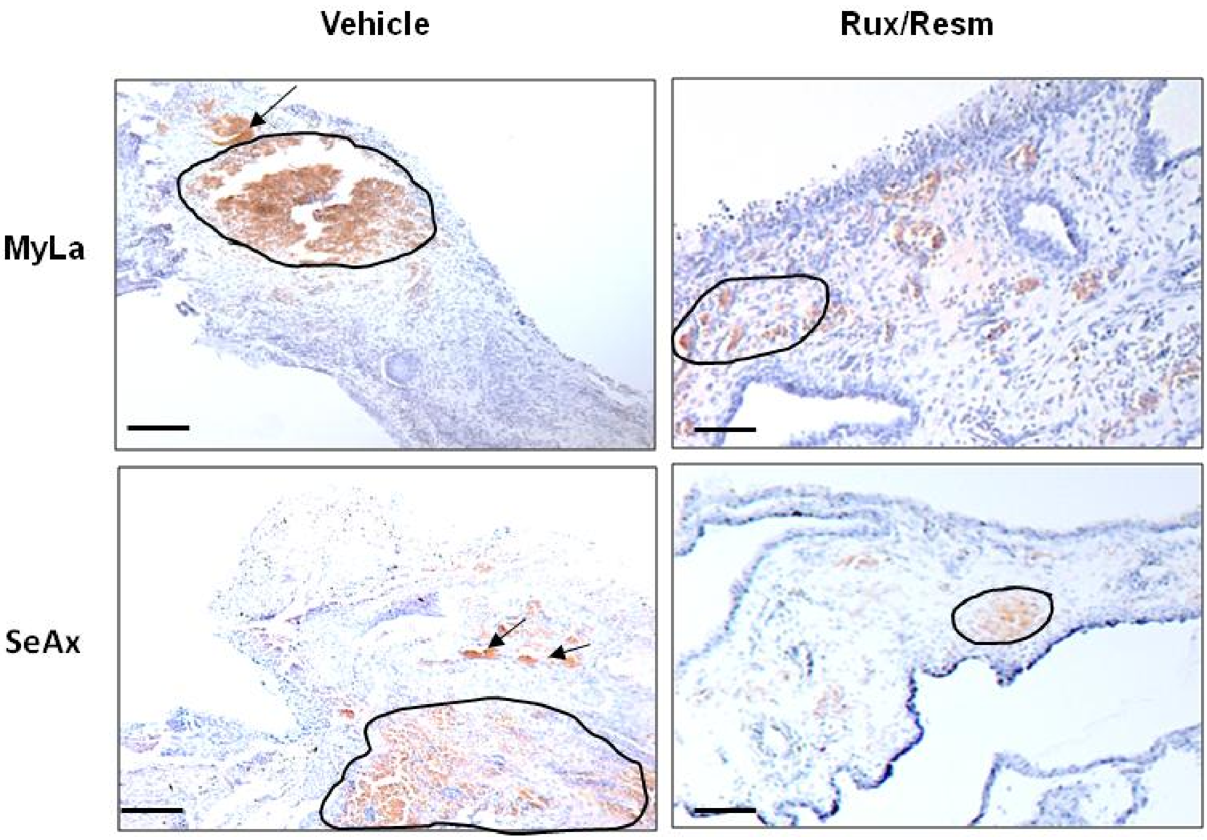

Supplementary Figure S1: Determination of efficiency of Resminostat (Resm) and Ruxolitinib (Rux) to inhibit growth of CTCL xenograft. Immunohistochemical visualization of CD44 expression in MyLa/ SeAx cells forming primary tumor and outgrowing from primary tumor by disrupting the CAM upper epithelium (black demarcation). Intravasated CTCL cells in the blood vessels of the CAM are shown by arrows. Scale bar: 200 um

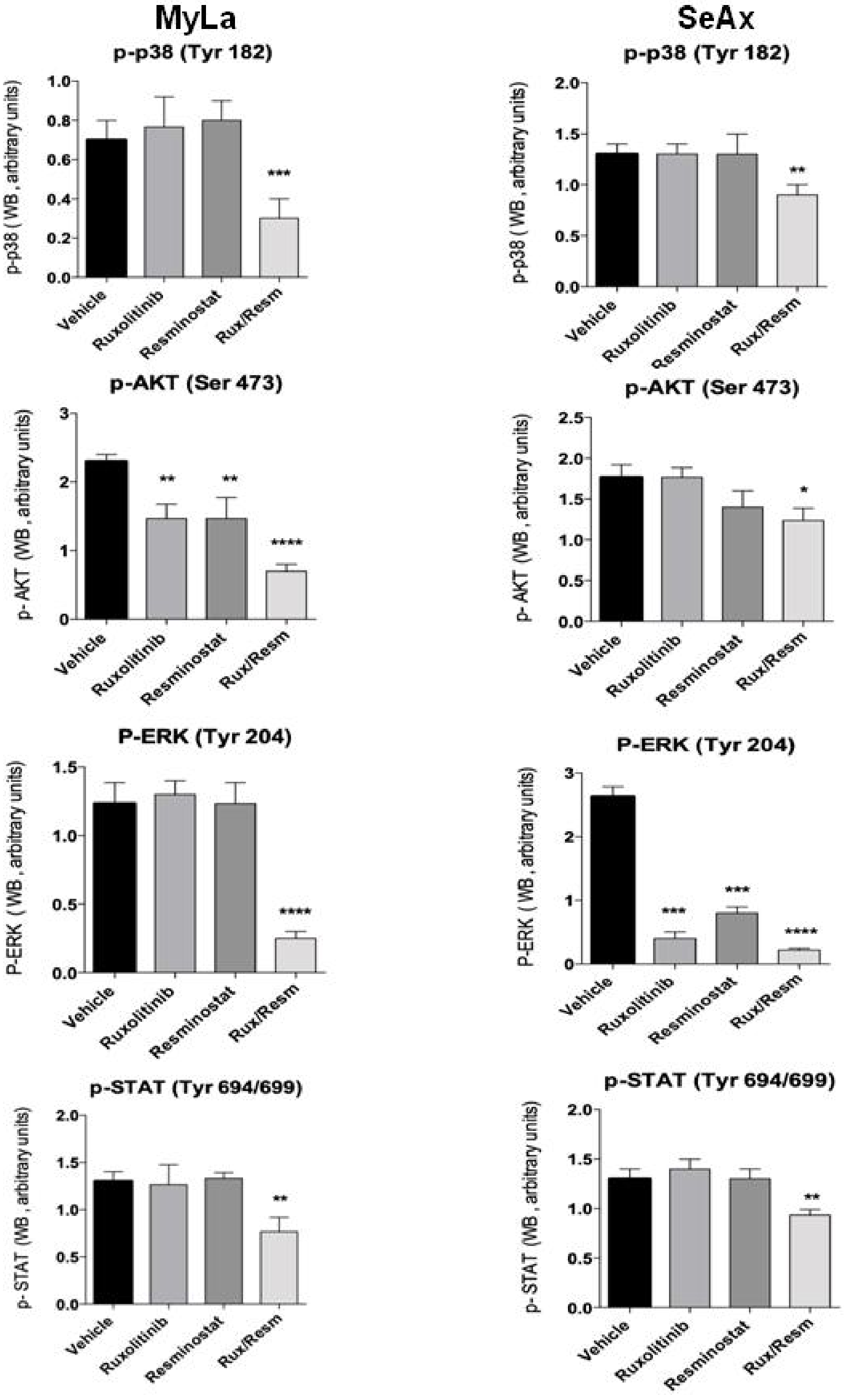
MyLa
SeAx
p-p38 (Tyr 182)
p-p38 (WB , arbitrary units)
p-AKT (Ser 473)
p- AKT (WB , arbitrary units)
P-ERK (Tyr 204)
P-ERK (WB , arbitrary units)
p-STAT (Tyr 694/699)
p- STAT (WB , arbitrary units)
Vehicle
Ruxolitinib
Resminostat
Rux/Resm
***
**
****
*

Supplementary Figure S2: Quantification of Western Blot analyses in CAM tumors. Images of wester blot were analysed by Image J software . Data show mean ± SEM from three (n = 3) independent experiments.

P values: * < 0.05, **< 0.005, ***< 0.001, ****< 0.0001

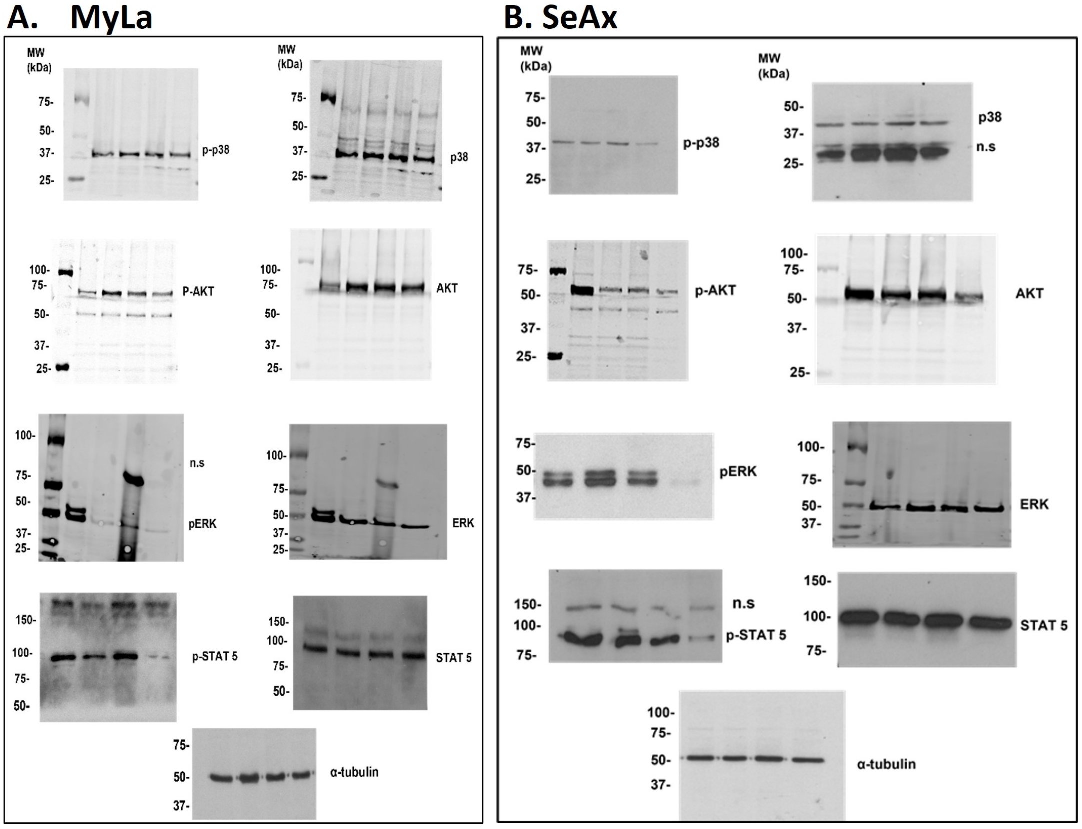

Supplementary FigureS3: Unprocessed images for Western Blot results corresponding to the Figure 7

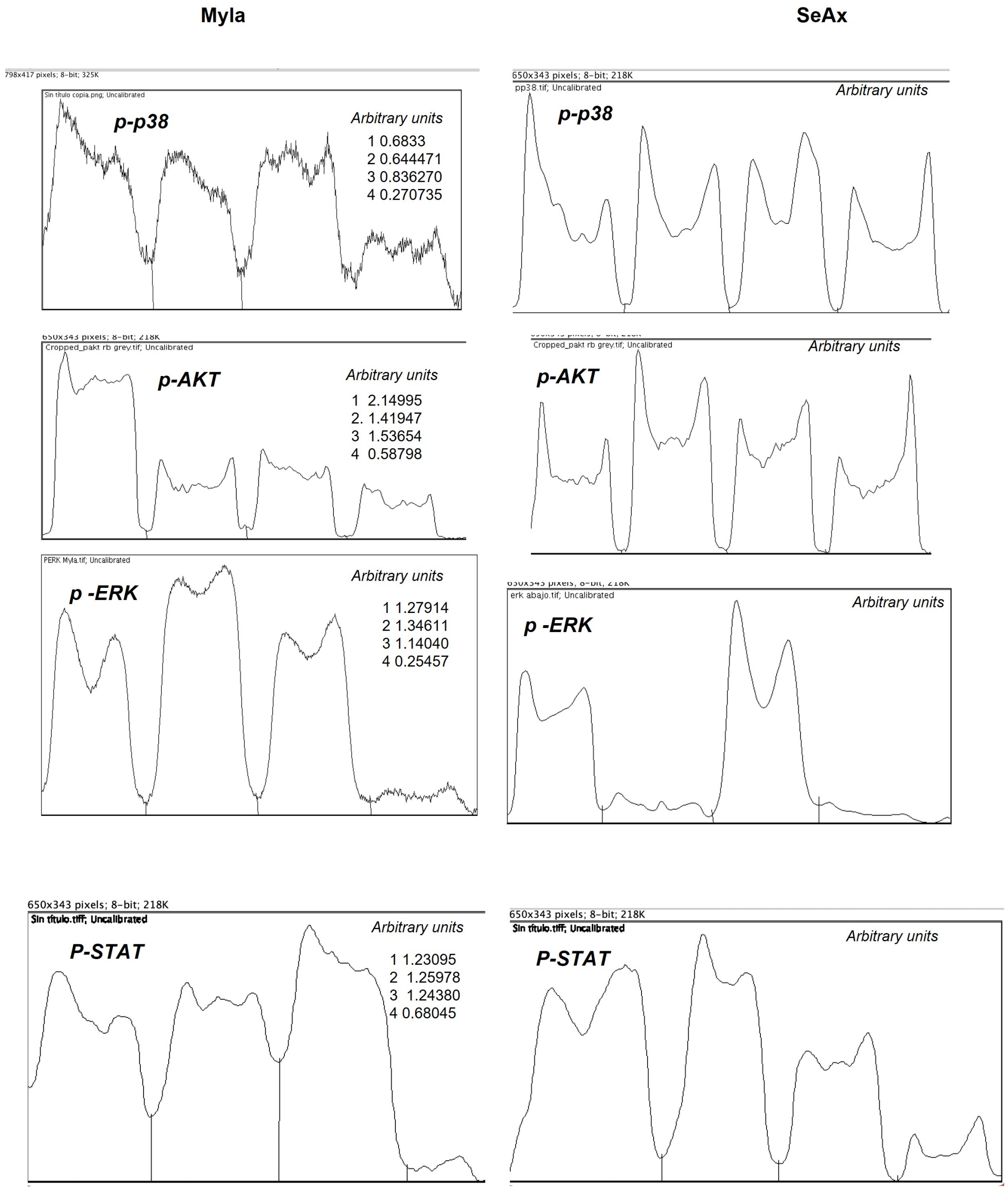

Supplementary Figure S4: Densitometry data generated for Western Blot results corresponding to the Figure 7 using Image J
